# Affective Norms for 4900 Polish Words Reload (ANPW_R): Assessments for Valence, Arousal, Dominance, Origin, Significance, Concreteness, Imageability and, Age of Acquisition

**DOI:** 10.3389/fpsyg.2016.01081

**Published:** 2016-07-18

**Authors:** Kamil K. Imbir

**Affiliations:** Faculty of Psychology, University of Warsaw Warsaw, Poland

**Keywords:** affective norms, duality of emotion, duality of activation, polish language, psycholinguistic indexes

## Abstract

In studies that combine understanding of emotions and language, there is growing demand for good-quality experimental materials. To meet this expectation, a large number of 4905 Polish words was assessed by 400 participants in order to provide a well-established research method for everyone interested in emotional word processing. The Affective Norms for Polish Words Reloaded (ANPW_R) is designed as an extension to the previously introduced the ANPW dataset and provides assessments for eight different affective and psycholinguistic measures of Valence, Arousal, Dominance, Origin, Significance, Concreteness, Imageability, and subjective Age of Acquisition. The ANPW_R is now the largest available dataset of affective words for Polish, including affective scores that have not been measured in any other dataset (concreteness and age of acquisition scales). Additionally, the ANPW_R allows for testing hypotheses concerning dual-mind models of emotion and activation (origin and subjective significance scales). Participants in the current study assessed all 4905 words in the list within 1 week, at their own pace in home sessions, using eight different Self-assessment Manikin (SAM) scales. Each measured dimension was evaluated by 25 women and 25 men. The ANPW_R norms appeared to be reliable in split-half estimation and congruent with previous normative studies in Polish. The quadratic relation between valence and arousal was found to be in line with previous findings. In addition, nine other relations appeared to be better described by quadratic instead of linear function. The ANPW_R provides well-established research materials for use in psycholinguistic and affective studies in Polish-speaking samples.

## Introduction

### Affective norms for verbal research stimuli

The affective nature of stimuli is an important issue when the consequences of emotions are the point of interest (Osgood et al., 1957; Russell, 2003). This applies to language and emotion relations. Therefore, with the use of Lang (1980) Self-assessment Manikin (SAM) scale, the Affective Norms for over 1000 English Words (ANEW: Bradley and Lang, 1999) dataset was introduced and stimulated the development of analogical datasets in numerous languages and cultures (for a review, see Table 1 in Riegel et al., 2015). The list of affective norms datasets is still growing because of the importance of such stimuli for all researchers interested in the interplay between language and emotion. Such datasets allow researchers to manipulate certain dimensions (e.g., valence) and to control for the potential effects of other dimensions (e.g., arousal, dominance, or concreteness). Different affective and psycholinguistic dimensions were demonstrated to shape the processing of stimuli in the mind (Citron et al., 2016). Taking this into account, all classical and some additional measures were included in the Affective Norms for Polish Words Reload (ANPW_R) dataset. The number of words assessed in the ANPW_R was increased in order to provide the biggest datasets among other word norms in the Polish language. In the next two sections, the importance of the affective and psycholinguistic dimensions included in the ANPW_R shown in previous research is described in detail.

**Table 1:** **SAM scales descriptions**.

Valence of experiences: negative vs. positive *Znak doznań: Negatywny kontra Pozytywny*	The first picture shows a person who is clearly distressed—relevant experiences could include panic, irritation, disgust, despair, defeat, or crisis. The last pictures shows an individual who is obviously elated—relevant experiences could include fun, delight, happiness, relaxation, satisfaction, or repose. The remaining pictures depict intermediate states.
Intensity of experiences: Tranquility vs. Excitation *Intensywność doznań: Spokój kontra Ekscytacja*	The first pictures shows an individual who is very calm, almost sleeping—relevant states could include relaxation, tranquility, idleness, meditation, boredom, or laziness. The last picture shows an individual who is bursting with arousal—relevant states could include excitation, euphoria, excitement, rage, agitation, or anger.
Sense of dominance: Being under control vs. Controlling *Odczucie dominacji: Bycie pod kontrolą kontra Kontrolowanie*	The first picture shows an individual who feels a lack of control and agency—relevant states could include subordination, intimidation, subjugation, withdrawal, submission, or resignation. The last picture shows a person who is dominant and in control of the situation—relevant states could include control, influence, being important, dominant, recognized, or decisive.
Origin of experience: from Heart vs. Reason *Pochodzenie doznań: z Serca kontra Rozumu*	The first picture shows an individual who is overwhelmed with appeals from the heart—words that could represent these experiences include being beside oneself, complete commitment, full engagement, impulsivity, spontaneity, lack of hesitation. The last picture shows a person who is under the sway of the mind, who is reflective—words that could be used to represent this state include feelings that result from contemplation, planning, consideration, prediction, choices, or comparisons.
Significance of experience: Insignificant vs. Significant for the individual *Waga doznań: Nieważne kontra Ważne dla człowieka*	The first picture shows a person whose current experience is not significant to his goals, plans, and expectations—his experience could be referred to using words such as trivial, gone unnoticed, fleeting, inconsequential, insignificant, unimportant. The last picture shows a person who is experiencing something very important to his goals, plans, and expectations—his experience could be referred to with words such as vitally important, significant, turning-point, consequential, meaningful, decisive.
Concreteness *Stopień konkretności lub abstrakcyjności słowa*	The words describe different things, conditions, actions, and features. Some are related to existing real objects such as house, tree, watermelon, carrots, or cat. Others, in turn, represent ideas that are born in our heads, such as justice, loyalty, goodness, thought or forecast. Think for a moment and indicate how, in your opinion, you associate the words presented with something concrete and tangible, which ones actually describe existing objects and things, and which are related to abstract ideas and thoughts.
Imageability *Na ile łatwo wyobrazić sobie obiekt lub stan opisywany przez słowo*	Words differ in how much they affect our senses. Some of them are hard to imagine or it takes a long time and requires a lot of effort to imagine them. On the other hand, some others capture the imagination and almost immediately the images associated with them appear in front of our eyes. Try to assess the extent to which the word is easy to imagine and associate with live images.
Subjective age of acquisition *Wiek w jakim człowiek uczy się danego słowa*	People are starting to learn words like “mom” or “dad”, and it will take some time before they will be able to write “Pan Tadeusz” [*famous Polish XIX century book written as a poem*]. Try to estimate how old is the person who learns the word. Think for a moment and enter next to the word the age at which more than half of people (children or adolescents) use this word.

### Affective qualities of stimuli: valence, dominance, origin, arousal, and subjective significance

Valence is the most intuitive property of an affective state (Kagan, 2007) and describes the pleasantness vs. the unpleasantness of feelings toward an object (Lang, 1980; Russell, 2003). This determines many of the processes in the cognitive domain ranging from memory modulation during stress (Smeets et al., 2006) to associations with vertical positions (Meier and Robinson, 2004), found to be up for positively valenced words but down in the case of negatively valenced stimuli. In addition, many electroencephalography (EEG) studies have shown that valence modulates cortical correlates of word processing (e.g., Citron, 2012; Kaltwasser et al., 2013; Imbir et al., 2015a). Norms collected for valence dimensions are the most reliable in terms of stability when assessed in test–retest and split-half estimation methods (c.f. Soares et al., 2012; Montefinese et al., 2014; Imbir, 2015a; Riegel et al., 2015).

Much less experimental work has been performed with the dominance dimension (c.f. Fontaine et al., 2007; Moors et al., 2013; Imbir, 2015a), which represents a measure of control toward perceived feelings evoked by stimuli, and varies from being under the influence of affect to being in charge of controlling ourselves. Dominance has also been operationalized in different ways in several studies. For example, Moors et al. (2013) used power or control (Fontaine et al., 2007) as an example of the dominance dimension (ranging from weak/submissive to strong/dominant). Dominance dimension, as well as valence and arousal, was found to reflect brain activity connected with current mood in a more coherent way than the traditional approach in mood description based on discrete emotional states (e.g., Wyczesany and Ligeza, 2015).

**Figure 1:**
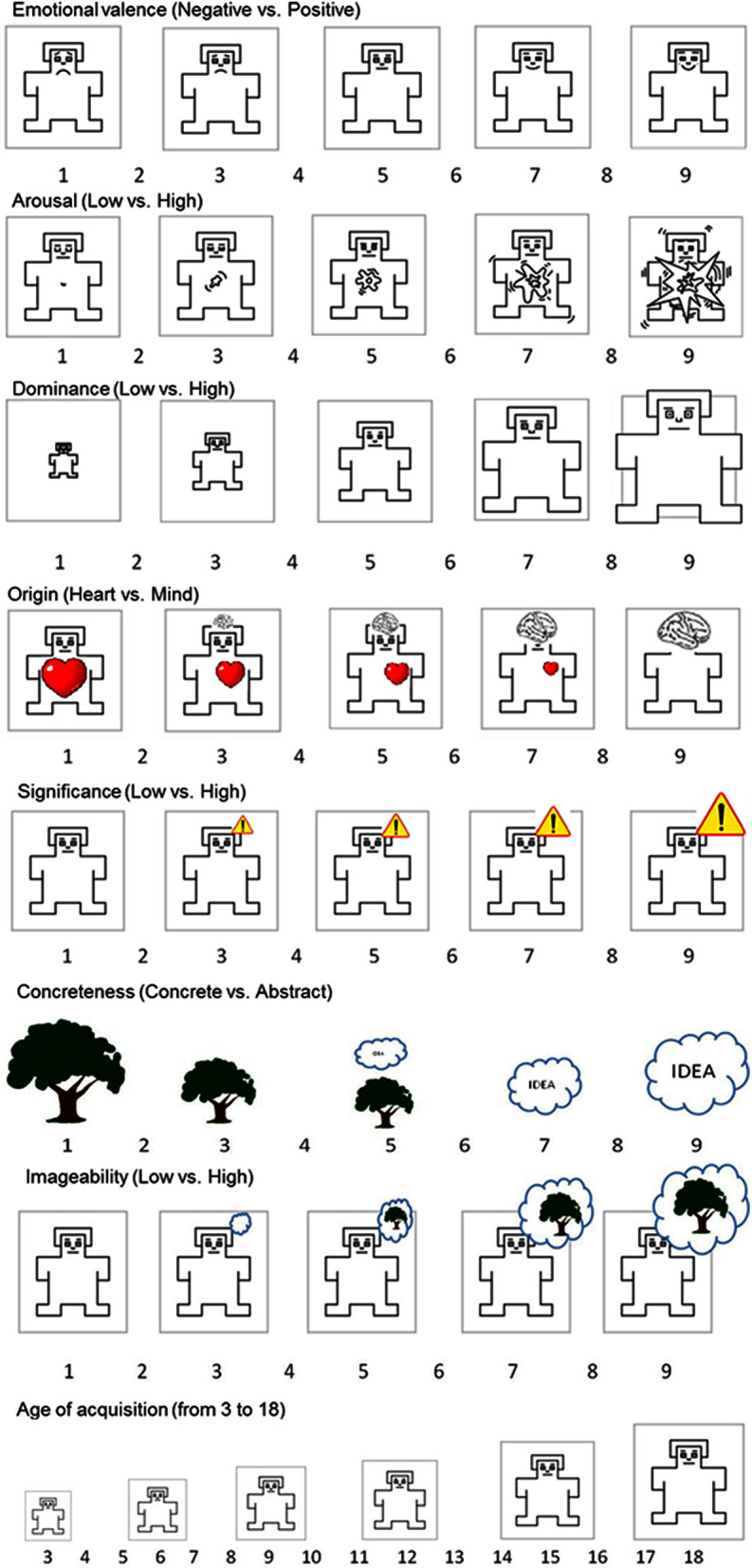
**Self-Assessment Manikin (SAM)**.

Last, the origin dimension, recently introduced by Jarymowicz and Imbir (2015), is the purely affective quality of stimuli. This represents the duality-of-mind-based distinction between two mechanisms of affective reaction formation. The SAM scale (Imbir, 2015a; c.f. Figure 1) consists of a bimodal scale representing the perceived origination of feelings from the heart or from the mind. The heart metaphor describes states that are automated and require fewer cognitive operations. Automatic emotional states appear to be spontaneous, quick and subjectively certain. In the formation of these states, a biological value criterion of evaluation (Damasio, 2010) is very important. The mind metaphor is defined as feelings that are deliberative, requiring a lot of cognitive operation, thus not spontaneous, but resulting from careful consideration. Such consideration is subjectively not free of doubt (due to the underlying multidimensional appraisals) and based on evaluative standards (Reykowski, 1989), representing verbalized criteria of evaluation. The original concept derives from the duality-of-mind theories perspective (for a review, see Gawronski and Creighton, 2013) and describes engagement of the mental system in the formation of the affective state (Automatic or Reflective Evaluating System: c.f. Jarymowicz and Imbir, 2015). Although origin is newly proposed, some experimental results show that it is worth investigating its consequences for cognition. For example, origin was found to modulate cognitive control in the Emotional Stroop Task and the Antisaccade Task (Imbir and Jarymowicz, 2013), making it hard to maintain control after automatic-originated (both negatively and positively valenced) words or sentences presentation. Other results concerning the scope of attention suggest that reflective-originated stimuli widen while automatic-originated stimuli narrow the scope in the visual field measured with the detection of stimuli that appear closer to or more distant from a center of visual field (Imbir, 2013). In addition, electrophysiological data (Imbir et al., 2015a) indicate that origin is useful in describing the mechanisms of emotional word processing and producing differences in amplitudes of evoked potentials that are independent from previously discovered effects of valence, arousal, frequency of use in language and concreteness. The SAM scale, developed to measure origin, appears to be a stable and reliable method of assessing this dimension (Imbir, 2015a).

Arousal is defined as an energetic reaction to stimuli varying from calm (sleep, no activation) to completely excited (extreme activation). In other words, arousal describes the energetic side of an affective state at a particular time and is sometimes referred to as the intensity or energy level. This energy expresses the degree of excitement or activation an individual feels toward a given stimulus (Lang, 1980); thus, the arousal level can be treated as a property of the stimulus that influences the current affective state (Russell, 2003). Arousal was found to modulate flanker competition in the flanker task (Freitas et al., 2007; Kuhbandner and Zehetleitner, 2011; Imbir, 2015b), cognitive control in the Emotional Stroop Task (e.g., Nigg, 2000; McKenna and Sharma, 2004) and electrophysiological correlates of word processing (e.g., Hofmann et al., 2009). Arousal best describes activation mechanisms for simple processes that do not require much cognition (Epstein, 2003) and was found to disturb high-order systematic processing (Kahneman, 2003, 2011) and to switch the balance between experimental and rational minds more toward the experimental one (Epstein, 2003).

Taking into account the duality-of-mind perspective, the question arises: what is the activation mechanism for rational and systematic effortful processing? This ought to be based on conscious attitudes toward stimulation concerning the significance of a situation in the context of subjective goals and expectations (Imbir, 2016a). From that point of view, subjective significance (Imbir, 2015a) was proposed and operationalized in the SAM scale analogous to arousal SAM (Lang, 1980). Some data suggest that subjective significance modulates the way in which arousal impairs cognitive control in the Emotional Stroop Test. Reaction latencies for highly arousing stimuli were shorter for low and high subjective significant words in comparison to words of medium significance (Imbir, 2016b). Subjective significance may be compared to impact operationalized for picture stimuli (Ewbank et al., 2009). Impact is defined as a visual media–related term describing that a certain stimulus has the potency to influence people, catch their attention and be remembered. Both concepts refer to the ability of stimulation to cause an intense reaction. Such intensity is analogous to arousal but engages more conscious- and more subjective-based processes, and thus should be considered in the dual-mind perspective as the reflective aspect of the intensity of the reaction to stimuli. Pictures of high-impact dimension values were found to be responsible for increased amygdale activation, compared to neutral, and low-impact stimuli (Ewbank et al., 2009). Another concept close to subjective significance is salience (e.g., Kahnt and Tobler, 2013), which describes the importance of outcomes. Considering decision making and risk, gains and losses associated with options given are different in valence but similar in salience. This means that people perceived some outcomes as important in comparison to neutral outcomes that are perceived as non-salient. Salience itself is not a quality of stimuli but the relation between stimuli in a task that requires decision making. Salience was found to modulate the neural response in decision-making procedures (c.f. Kahnt and Tobler, 2013). Since the concept of rational mind activation is a rather new one in psychology (see Imbir, 2016b), ANPW_R provides a unique measure of this property of stimuli.

### Psycholinguistic qualities: concreteness, imageability, and subjective age of acquisition

Some qualities of words provided in the ANPW_R are not affective, but they may have a potential impact on word processing (Moors et al., 2013). The decision on their inclusion was based on the potential role for an alternative explanation for affective dimension outcomes in order to provide a comprehensive dataset for researchers. The concreteness dimension describes the type of stimuli in the case of words related to concrete vs. abstract objects. In other words, concreteness refers to the ability to see, hear, and touch something (Bird et al., 2001). Concreteness was measured for verbal stimuli several times (e.g., Kanske and Kotz, 2010; Ferré et al., 2012; Montefinese et al., 2014; Hinojosa et al., 2016) and was found to modulate the event-related potential (ERP) correlates of emotional word processing (c.f. Kanske and Kotz, 2007; Barber et al., 2013; Palazova et al., 2013). What is more, concreteness interplays with valence in the way that abstract words were found to be perceived in a more valenced way than concrete words (c.f. Vigliocco et al., 2014).

Imageability represents the degree of how easy it is to imagine the objects or states represented by the stimulus (Bird et al., 2001). From a theoretical point of view, imageability could be similar to concreteness, but imageability involves not only the cognitive aspect of stimuli concreteness perception but also the active imagination connected with mental representation creation and perhaps the number of interactions with word designates. Imageability has been measured for verbal stimuli several times (e.g., Bird et al., 2001; Cortese and Fugett, 2004; Võ et al., 2006, 2009; Janschewitz, 2008; Citron et al., 2014; Monnier and Syssau, 2014; Schmidtke et al., 2014; Riegel et al., 2015) and was found to be involved in word recognition processes (e.g., Davelaar and Besner, 1988) and memory (e.g., Sadoski and Paivio, 2001).

Subjective age of acquisition (AoA), which representsthe subjectively perceived difficulty of words, was found to be correlated with word frequency (Bird et al., 2001). High-frequency words tend to be learned early in life. Subjective age of acquisition has been measured in some affective norms studies (e.g., Moors et al., 2013; Warriner et al., 2013; Citron et al., 2014) and was found to be the most important factor determining word recognition response times, after frequency, length, similarity to other words and words onset (Kuperman et al., 2012). In addition, in a Dutch-speaking sample, frequency and AoA left no variance for imageability in visual word recognition (Brysbaert et al., 2000).

### Polish affective norms for datasets of words

Until now, only two datasets contain affective norms for Polish verbal stimuli (Imbir, 2015a; Riegel et al., 2015). The first dataset, the ANPW (Imbir, 2015a), provides the norms for six dimensions (valence, arousal, dominance, origin, subjective significance, and source) for 1586 Polish words and compound expressions collected from a large group of participants (more than 1600) with the use of a standard paper-and-pencil procedure. The ANPW list was based on ANEW (Bradley and Lang, 1999) translated and extended by additional words considered good representations of extreme origin and subjective significance values. The second dataset in the Polish language is the Nencki Affective Word List (NAWL; Riegel et al., 2015), a dataset that provides assessments for valence, arousal and imageability for 2902 words assessed by 266 Polish participants in a computerized procedure. The NAWL is a Polish adaptation of the Berlin Affective Word List-Reloaded (BAWL-R; Võ et al., 2009). As a supplement to the NAWL, assessments of compliance with basic emotions (happiness, anger, sadness, fear, and disgust) were developed (Wierzba et al., 2015).

### Aim and hypothesis

The motivation for introducing the ANPW_R was to provide research materials for scientists interested in the interplay between language and emotions (e.g., Citron, 2012; Kaltwasser et al., 2013; Imbir et al., 2015a). The areas of interest for affective norms for words are not limited to emotional scientists but also extend to researchers interested in psycholinguistics, including more complex processes such as morphosyntactic processing (Martín-Loeches et al., 2012; Hinojosa et al., 2014; Díaz-Lago et al., 2015) or phonological processes during language production (Hinojosa et al., 2010; White et al., 2016). The main aim of the current work was to extend a recently introduced the ANPW (Imbir, 2015a) dataset to a greater number of words, as well as to assess the properties of stimuli using new scales such as concreteness and subjective age of acquisition. These two dimensions have never been assessed in Polish language normative studies for words. An additional aim was to check whether ratings collected with a low number of participants assessing a large number of stimuli are as reliable as the traditional paper-and-pencil procedure used with a large number of participants assessing a small number of stimuli.

The ANPW_R dataset was expected to be reliable (in terms of split-half estimates) and stable (in terms of correlation with the ANPW (Imbir, 2015a), a previously conducted normative study for a Polish language sample, for valence, arousal, dominance, origin, and significance, as well as correlations with the NAWL (Riegel et al., 2015) for valence, arousal, and imageability. In addition, a quadratic relation between valence and arousal (e.g., Ferré et al., 2012; Soares et al., 2012; Monnier and Syssau, 2014; Riegel et al., 2015), as well as dominance and arousal (Montefinese et al., 2014), was expected. Furthermore, in light of the literature (e.g., Ferré et al., 2012; Monnier and Syssau, 2014; Montefinese et al., 2014; Riegel et al., 2015), gender differences in female and male assessments of words were expected for affective and psycholinguistic variables, especially more polarized assessments for women for valenced stimuli.

## Methods

### Participants

The study involved 400 participants (200 females) aged from 18 to 32 (*M* = 21.89, *SD* = 1.91), students from different Warsaw universities and colleges of natural sciences (32%, *N* = 128), social sciences (excluding psychology students) and humanities (36%, *N* = 144) and technical sciences (32%, *N* = 128). The proportion of sexes across faculty types was balanced (50% female in each case) in order to avoid any sex bias over affective evaluations. Participation was voluntary in nature and was rewarded by a small prepaid gift card (about €20 each). Participants were recruited via Internet faculty sites and via traditional posters placed indifferent departments. Participants provided informed consent to participate; written consent was not collected as the participants were assured anonymity. Participants provided informed consent via the Internet to the lab member who recruited the participants and was documented in a research diary. This procedure was suggested by the bioethical committee that approved the research. No personal data were collected from the participants. The design, the experimental conditions and the consent procedure for this study were approved by the bioethical committee of the Maria Grzegorzewska University. Contact with participants was maintained via email. After the assessments were completed, a single laboratory meeting took place.

### Materials and design

#### Self-assessment manikin (SAM) scales

To measure five affective as well as three psycholinguistic variables, the SAM scales were applied. In the case of the classical affective dimensions (valence, arousal, and dominance), the original Lang (1980) SAMs were used. To measure origin and subjective significance, both describing variables from the emotional duality model (Jarymowicz and Imbir, 2015) scales introduced in the ANPW (Imbir, 2015a) were used. To measure psycholinguistic variables (concreteness, imageability and subjective age of acquisition) three new SAM scales were created in order to assure formal similarities with affective ratings. Figure 1 presents SAMs used in the current study.

Because of the fact that some scales were easier to understand for naïve participants (e.g., valence, imageability) and some others could be more difficult (e.g., dominance, origin, significance), additional descriptions of scales were provided (c.f. Imbir, 2015a; submitted). Those descriptions explained in detail the meaning of the scales and provided examples of both ends of the scales. The words presented as examples were chosen in a manner that presented different aspects of each scale end. For example, in the case of origin, both automatic and reflective origins were exemplified by negative and positive instances. Table 1 presents descriptions of each scale used in the current study. Those for valence, arousal, dominance, origin, and subjective significance scales were identical as those used in the ANPW dataset creation (c.f. Imbir, 2015a).

#### List of 4905 polish words

The list of stimuli used in the current experiment was based on two main sources. First of all, 1586 words were taken from the ANPW (Imbir, 2015a). The aim of this decision was to estimate similarities in using different methods of obtaining affective ratings (classical paper and pencil used on a large number of participants, and the new method, based on a large number of assessments done by a much smaller number of participants (c.f. Moors et al.'s, 2013) and to collect new assessments for words of a psycholinguistic nature not included in the ANPW dimensions. The remainder of the words was taken from Moors et al. (2013) Dutch Affective Words Norms list of 4299 items translated into Polish. The 4299 words were presented in their original list in two different languages (Dutch and English translations), thus the computerized translation of the Google Translate engine was applied in the first stage. The algorithm was simple; the Dutch and English lists were translated separately into Polish and then compared in line with translation procedure. In 3270 cases, the Polish translation was the same in both lists, thus this was accepted as valid. The remaining 1029 words were carefully inspected by a bilingual person who specializes in the English language. Unfortunately, there was no person bilingual in Dutch and Polish available at the time of translation, thus at this stage, the Polish Google machine translations from Dutch and English, the English version of words and the Dutch part of speech (data provided in Moors et al., 2013) were used as the basis for further decisions. It appeared that in 678 cases, both computer translations from Dutch and English differed in Polish flexion (nouns and verbs have a lot of versions), so, translations were corrected to their base form and accepted. The remaining 351 cases were translated by an English language philologist who specializes in translations. In the final list of 4299 Polish words, 1057 duplicates were found (321 among the translations and 736 with comparison to the ANPW), thus only 3242 new words were added to the previously collected 1586 words. Other Polish words were included covering: some neutral terms (nouns describing actions) from earlier studies conducted by this author (*N* = 28), Polish vulgarisms (*N* = 5) and names of European or world states and nations (*N* = 44). All this comes to 4905 words included for assessment in the ANPW_R dataset. The whole list consists of 2907 nouns (59%), 1126 verbs (23%), 768 adjectives (15%), 44 adverbs (.8%), and 60 others (including two compound words expressions).

#### Questionnaires prepared

To make the assessments more accessible to participants, a computerized Excel spreadsheet questionnaire, similar to those used by Moors et al. (2013), was prepared. The whole questionnaire consisted of four different spreadsheets. The first explained the aim of the study, the importance of the results obtained and what was involved in completing the questionnaire. At this stage, the SAM scale was described in terms of its idea of emotional states presented in a non-word, pictorial nature that helps in intuitive judgments of feelings and current states. Participants were also informed that there would be a description of the scale provided in order to clarify the meaning of both ends of the scale. The required type of response to the words was described as placing numbers (from 1 to 9 in the case of seven different measures, and from 3 to 18 in the case of subjective age of acquisition) next to the assessed word. It was highlighted that this was a subjectively based validation, thus there was no question of responses being judged as “bad” or “good” answers. In addition, instruction was provided to encourage quick validation and to split the whole work into 5–7 short sessions, one a day each. Participants were also asked to leave empty spaces and not to assess words they do not know themselves. The second spreadsheet consisted of sociodemographic data (sex, age, number of years at university, department type). The third spreadsheet presented the training session. The SAM scale and its description were placed at the top of the page. Below this, three example words were placed (not included in the 4905 dataset) and the task was to evaluate them using the SAM scale. The last spreadsheet presented a SAM scale with its description and below a full list of the 4905 stimuli presented in a unique random order that was different for each participant. The SAM scale was visible at all times at the top of the spreadsheet during the assessment process, in order to provide a continuous reference point.

### Procedure

The task for the participants was to evaluate a list of 4920 words (15 were doubled in order to provide additional estimation of reliability (c.f. Imbir, 2015a) using a single SAM scale described in detail at the beginning of procedure. At the end of a week, the researcher sent some recruited volunteers the Excel spreadsheets to collect the assessments. Participants were instructed to perform the procedure at their own pace in short sessions over the whole week. They were asked to perform their assessments in a stable environment without any distractions. Confirmation of having fulfilled these procedure requirements was mandatory after sending the results back. In the following week, participants were invited to the laboratory to collect their reward. At this stage, all participants' questions were answered and the procedure was explained in detail. Interviews were also focused on checking that the procedure requirements had been fulfilled in order to establish whether any of the requirements had not been met. About 10 participants were excluded because they had not fulfilled the procedure requirements and their assessments were replaced by those of other, additional participants.

## Results

### Data treatment and analytic strategy

The first step was to enter data into the database. Only questionnaires from participants who had fulfilled the criteria of responding within 1 week and who did not report any abnormalities during their work were included. Then descriptive statistics [number of assessments (*N*), Mean (*M*), Standard Deviation (*SD*), Range (Min and Max values)] were calculated for each word, separately for each of the 8 scales. All analyses were carried out using IBM SPSS 22 statistical software. The Supplemental Material (Appendix 1) includes all values for valence, arousal, dominance, origin, significance, concreteness, imageability, and subjective age of acquisition assessments. Each word was rated by 400 participants. Each scale was assessed by 50 participants (25 females). Participants were instructed to leave words without an assessment in the case of words not familiar to them. The number of participants indicating that they did not know a certain word varied from 0 to 244 (*M* = 2.29, *SD* = 13.52). For that reason some ratings are calculated based on a lower number of assessments.

Data were analyzed in order to achieve: (1) the verification of the ANPW_R dataset reliability, (2) understanding of the impact on assessments of other factors, like participants' sex as well as, (3) verification of the relations between measured dimensions. First of all, the properties of measures were assessed with descriptive statistics. Secondly, to validate assessments collected in the current study, reliability, and stability of assessments was estimated with the use of four different approaches based on the current dataset (split-half correlations and doubled words in list assessments congruency) and earlier studies (congruencies in ratings for certain words between the ANPW_R and the ANPW or the NAWL). Also, sex differences were assessed with the use of *r*-Pearson correlations and ANOVA analyses in order to check if the perception of words in affective as well as psycholinguistic variables differs across genders. Finally, the relations between measures were analyzed with use of linear (*r*-Pearson correlation) as well as curvilinear (Regression analyses) models.

### Descriptive statistics

Table 2 presents descriptive statistics for the assessments of all affective and psycholinguistic variables used and the lexical dimensions such as number of letters in word (length) and frequency estimations based on two sources: Subtlex_pl, dataset created on the basis of movies and television programs subtitles (Mandera et al., 2014) and Kazojć (2011) dataset of huge literature, electronic texts and web pages collections.

**Table 2:** **Summary of variables included in the word list with means (***M***), standard deviations (***SD***) and ranges for all participants**.

	**Affective ratings**			**Number of collected ratings**		
	***M***	***SD***	**Range**	***M***	***SD***	**Range**
Valence	5.01	1.29	1.44–8.40	49.71	1.86	16–50
Arousal	4.08	0.86	2.06–7.18	49.77	1.35	26–50
Dominance	5.12	1.00	2.04–8.10	49.70	1.87	16–50
Origin	5.44	0.83	2.28–7.78	49.74	1.56	26–50
Significance	3.83	0.86	1.86–7.02	49.84	1.13	21–50
Concreteness	4.13	1.65	1.44–7.72	49.63	2.13	14–50
Imageability	6.29	1.21	2.53–8.48	49.55	2.56	10–50
Age of Acqisition	9.13	1.74	3.62–14.06	49.78	1.51	21–50
LN Frequency (Subtlex_pl)	5.75	2.35	1.10–15.16			
LN Frequency, (Kazojć, 2011)	5.93	2.33	0.69–14.72			
Number of letters	7.61	2.83	2–22			

**Figure 2:**
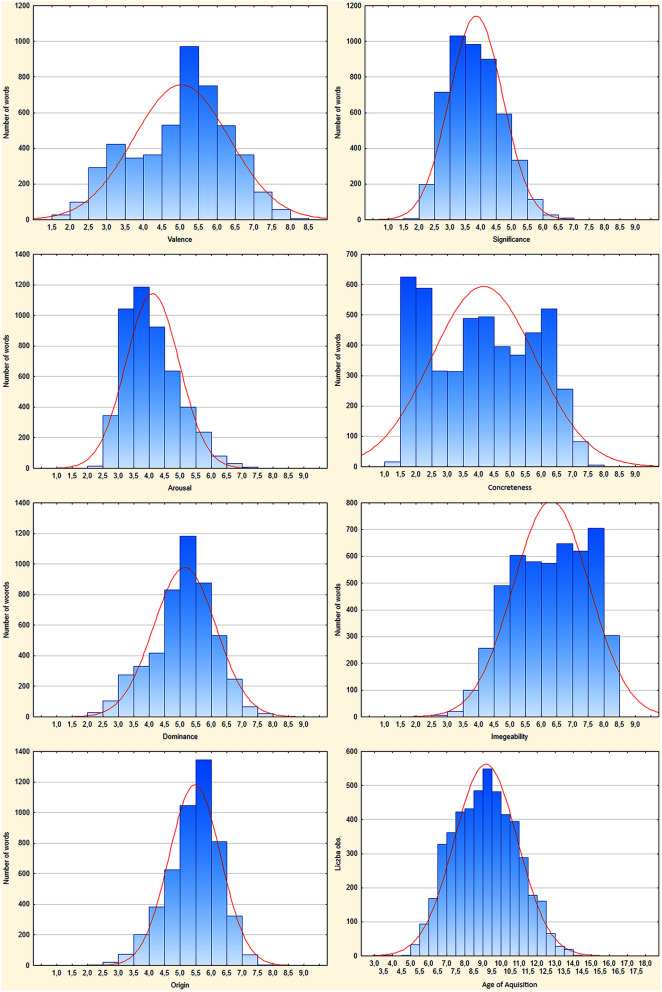
**Histograms presenting number of words assessed in 0.5 intervals from 1 to 9 of SAM scales**.

Figure 2 shows the distributions of eight measures. The distributions for valence and concreteness are bimodal, while imageability is flat and biased toward high scale values. Dominance meets the best approximate normal distribution centered over the middle of the scale. In the case of arousal and subjective significance the distribution is approximately normal with a negative bias (toward low scale values), whilst in the case of origin, the approximately normal distribution is positively biased (toward high scale values).

**Figure 3:**
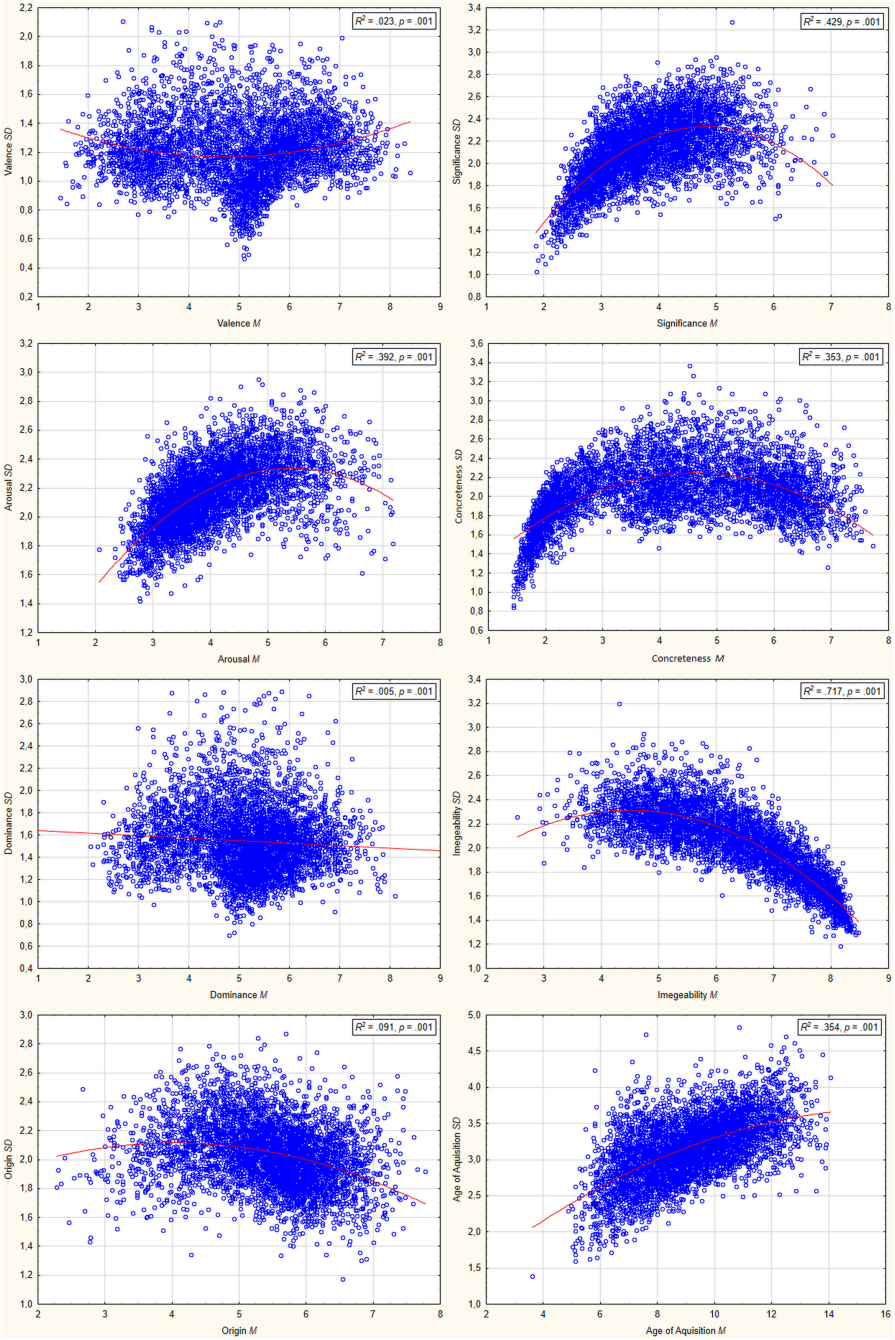
**Means (***M***) plotted against standard deviations (***SD***) and function best fitted to the data**. In right top corner of each distribution *R*^2^-value for plotted function.

Figure 3 shows homogeneity of ratings in terms of means plotted against their standard deviations for each measure applied in the ANPW_R. Additionally, regression lines with *R*^2^- and *p*-values for each case are provided. Ratings' distribution in *M* × *SD* space gives us information concerning to what extent assessments were congruent. It is especially important for neutral / moderate (around middle of the scale) assessments that may be the result of (a) neutral or moderate properties of the stimulus when *SD* is low or (b) incongruent assessments, when some participants rate the stimulus as low whereas other participants rate it as high in certain measures. For example, in the valence dimension among neutral stimuli some have low *SD* whereas others have high *SD*-values. In most of the cases (apart from dominance) the relationships plotted were better explained by a quadratic unction rather than a linear (in terms of bigger *R*^2^ and significant *R*^2^ change). The most frequent relationship observed is reversed “U” shaped relation, suggesting that neutral / moderate stimuli are in fact more incongruent in assessments. This is not surprising, taking into account that a word can obtain an extreme mean value only when most of the assessments are as extreme as mean itself is, thus extreme stimuli are more congruent than moderate ones. Surprisingly, in the case of valence, the relation is “U” shaped, not reverse “U” shaped. There is a group of neutral stimuli that were very low in *SD*-values (c.f. Figure 3). A similar pattern was found in the case of an Italian adaptation of ANEW (Montefinese et al., 2014).

### Reliability of measurement

To measure reliability two types of estimations were applied. The first was the split-half method based on splitting the entire number of into two separate subsets. The split was based on the participants' numbering (odd or even) with respect to gender balance for both subsamples. The second was introduced in the ANPW (Imbir, 2015a) dataset and was based on including into the assessed words list some randomly chosen doubled stimuli. In the current study 15 words were repeated and placed in random positions in the 4905 words list. Participants were not aware that some words were repeated and afterwards nobody indicated that fact. This was probably because participants assessed words on different days during the week.

With respect to the split-half estimate, the Pearson correlations were applied. Due to splitting the whole dataset into two halves the Spearman–Brown formula was applied to adjust correlations due to the lower—in comparison to the whole research probe collected—number of participants in both subsets. In all cases the correlations were high and significant, varying from 0.828 (0.906 with S-B formula adjustment) for origin to 0.979 (0.986) for valence. Table 3 presents the pattern of correlation for each of the eight measures.

**Table 3:** **Reliability estimations for each variable**.

	**Reliability and stability**				**Female–male ratings^d^**
	**Split-half estimation^a^**	**Split-half estimation Spearman-brown adjustment^a^**	**ANPW (Imbir, 2015a) correlations^b^**	**NAWL (Riegel et al., 2015) correlations for 1274 words^c^**	
Valence	0.973	0.986	0.927	0.947	0.964
Arousal	0.841	0.914	0.762	0.732	0.858
Dominance	0.868	0.929	0.844		0.909
Origin	0.828	0.906	0.763		0.843
Significance	0.852	0.92	0.738		0.749
Concreteness	0.959	0.979			0.944
Imageability	0.941	0.97		0.827	0.925
Subjective age of acquisition	0.932	0.965			0.907

a *Split-half correlations (r-Pearson's) estimation for all words and Spearman–Brown adjustments;*

b *Correlations (r-Pearson's) with 1586 ANPW dataset;*

c *Correlations (r-Pearson's) with 1274 words from NAWL dataset (Riegel et al., 2015);*

d *Correlations (r-Pearson's) between female and male assessments*.

To measure whether 15 repeated random words were assessed in the same way, the ANOVA analysis was applied. Repetition (first vs. second) and paired words' number (1–15) were treated as within-subject factors. Eight different (one for each dimension measured) ANOVAs were conducted. Only the main effects of repetition interesting from a theoretical point of view will be presented here. In all cases word pairs differed significantly from one another, but this is an obvious effect, thus would be omitted. In all cases ANOVA analysis showed no significant differences between the first and second assessment of 15 repeated words for valence: *F*_(1, 49)_ = 1.36, *p* = 0.25, η^2^ = 0.027; arousal: *F*_(1, 48)_ = 1.45, *p* = 0.23, η^2^ = 0.029; dominance: *F*_(1, 49)_ = 0.06, *p* = 0.81, η^2^ = 0.001; origin: *F*_(1, 49)_ = 0.48, *p* = 0.5, η^2^ = 0.01; significance: *F*_(1, 48)_ = 0 2.1, *p* = 0.15, η^2^ = 0.042; concreteness: *F*_(1, 49)_ = 1.41, *p* = 0.24, η^2^ = 0.029; imageability: *F*_(1, 49)_ = 0.27, *p* = 0.6, η^2^ = 0.006; or the subjective age of acquisition: *F*_(1, 49)_ = 0.44, *p* = 0.5, η^2^ = 0.009.

### Stability of measurement

To measure the stability of affective ratings, the Pearson correlations were applied for words from the ANPW (*N* = 1585) repeated in the ANPW_R for five affective variables measured in both studies: valence, arousal, dominance, origin, and significance. Both studies used different methodologies of assessment collection—paper-and-pencil was run over a huge sample in the ANPW case and computerized method was used over a much smaller sample in the ANPW_R case. It appears that both methods generated very similar results. All correlations were significant and assessments correlate from 0.738 in the case of the subjective significance scale to 0.927 in the case of the valence scale.

Correlation analyses with another existing Polish Word norms dataset of 2902 words (NAWL: Riegel et al., 2015) including valence, arousal and imageability assessments were performed. It appears that 1274 words from the NAWL were included in the ANPW_R, so for this subset stability of ratings was checked. Correlations were high and varied from 0.947 for valence, 0.732 for arousal to 0.827 for imageability. Table 3 presents obtained results for both existing datasets and the ANPW_R dataset.

### Sex differences

In order to compare perception of affective words included in the ANPW_R across both sexes two methods were applied. The first was a Pearson correlation of ratings given by females and males. The affective ratings were calculated separately for all women and men participating in the final data. All correlations were significant (*p* < 0.001) and varied from 0.749 for significance to 0.964 in the case of valence. The last column in Table 3 presents results for each dimension.

The second approach used to measure gender differences was to search for differences in average ratings for all of the eight measured dimensions. To do so, eight different analyses of variance (one for each dimension) were applied. Sex was treated as a within-words factor and valence was treated as a between-words factor. Valence was divided into three categories based on sentence average scores—negative: 1–4; neutral: 4–6 and positive: 6–9 (c.f. Ferré et al., 2012; Monnier and Syssau, 2014)—and used in each analyses as the easiest and most intuitive dimension to search for more subtle effects. Such an approach was used earlier to assess gender differences (e.g., Monnier and Syssau, 2014). Table 4 presents the mean assessments for female and male participants in case of each analyzed dimensions. Table 5 presents results of ANOVA analyses. Valence effects were checked with *post-hoc* Scheffé test. Significant (*p* < 0.05) differences between valence categories are shown in separate column.

**Table 4:** **Mean assessments for female and male participants in case of each analyzed dimension**.

		**Negative**	**Neutral**	**Positive**	**Total**
		**(*N* = 1186)**	**(*N* = 2600)**	**(*N* = 1119)**	**(*N* = 4905)**
Valence	Female	3.22 (0.94)	5.08 (0.73)	6.54 (0.89)	4.96 (1.41)
	Male	3.55 (0.85)	5.19 (0.60)	6.36 (0.78)	5.06 (1.20)
Arousal	Female	5.08 (0.93)	3.88 (0.73)	4.24 (0.89)	4.25 (0.95)
	Male	4.64 (0.80)	3.61 (0.65)	3.86 (0.74)	3.92 (0.83)
Dominance	Female	4.20 (1.09)	5.35 (0.78)	6.03 (0.81)	5.23 (1.08)
	Male	4.21 (0.99)	5.10 (0.74)	5.67 (0.82)	5.02 (0.97)
Origin	Female	5.29 (0.75)	5.88 (0.67)	5.26 (1.02)	5.60 (0.84)
	Male	4.90 (0.80)	5.58 (0.73)	5.03 (1.06)	5.29 (0.89)
Significance	Female	4.33 (0.88)	3.63 (0.94)	4.78 (1.16)	4.06 (1.09)
	Male	3.63 (0.63)	3.36 (0.64)	4.08 (0.80)	3.59 (0.74)
Concreteness	Female	4.56 (1.75)	3.21 (1.69)	4.40 (2.06)	3.81 (1.91)
	Male	5.05 (1.23)	4.00 (1.34)	4.89 (1.47)	4.45 (1.44)
Imageability	Female	6.09 (1.19)	6.66 (1.34)	6.46 (1.37)	6.48 (1.33)
	Male	5.73 (1.01)	6.31 (1.13)	6.02 (1.19)	6.10 (1.14)
Subjective age of acquisition	Female	9.73 (1.79)	9.08 (2.03)	8.67 (1.98)	9.14 (2.00)
	Male	9.51 (1.47)	9.08 (1.59)	8.78 (1.51)	9.11 (1.56)

**Table 5:** **Valence, Sex, and interaction of valence and Sex impact on each analyzed dimension**.

**Dimension**	**Main effect of sex**	**Main effect of valence**	**Significant contrasts (post-hoc Scheffé test) for Valence groups differences**	**Interaction of sex and valence**
Valence	*F*(1, 4902) = 233.02,	*F*(2, 4902) = 4952.4,	Neg-Neu (*p* = 0.001),	*F*(2, 4902) = 551,75,
	*p* = 0.001, η2 = 0.045	*p* = 0.001, η2 = 0.669	Neg-Pos (*p* = 0.001),	*p* = 0.001, η2 = 0.184
			Neu-Pos(*p* = 0.001)	
Arousal	*F*(1, 4902) = 2377.84,	*F*(2, 4902) = 971.43,	Neg-Neu (*p* = 0.001),	*F*(2, 4902) = 58.17,
	*p* = 0.001, η2 = 0.327	*p* = 0.001, η2 = 0.284	Neg-Pos (*p* = 0.001),	*p* = 0.001, η2 = 0.023
			Neu-Pos(*p* = 0.001)	
Dominance	*F*(1, 4902) = 912.17,	*F*(2, 4902) = 1197.13,	Neg-Neu (*p* = 0.001),	*F*(2, 4902) = 235.8,
	*p* = 0.001, η2 = 0.157	*p* = 0.001, η2 = 0.328	Neg-Pos (*p* = 0.001),	*p* = 0.001, η2 = 0.088
			Neu-Pos(*p* = 0.001)	
Origin	*F*(1, 4902) = 1710.5,	*F*(2, 4902) = 385.46,	Neg-Neu (*p* = 0.001),	*F*(2, 4902) = 34.26,
	*p* = 0.001, η2 = 0.259	*p* = 0.001, η2 = 0.136	Neu-Pos(*p* = 0.001)	*p* = 0.001, η2 = 0.014
Significance	*F*(1, 4902) = 2699.92,	*F*(2, 4902) = 607.25,	Neg-Neu (*p* = 0.001),	*F*(2, 4902) = 229.86,
	*p* = 0.001, η2 = 0.355	*p* = 0.001, η2 = 0.199	Neg-Pos (*p* = 0.001),	*p* = 0.001, η2 = 0.086
			Neu-Pos(*p* = 0.001)	
Concreteness	*F*(1, 4902) = 9.05,	*F*(2, 4902) = 324.53,	Neg-Neu (*p* = 0.001),	*F*(2, 4902) = 102.96,
	*p* = 0.001, η2 = 0.37	*p* = 0.001, η2 = 0.117	Neg-Pos (*p* = 0.036),	*p* = 0.001, η2 = 0.04
			Neu-Pos(*p* = 0.001)	
Imageability	*F*(1, 4902) = 2384.87,	*F*(2, 4902) = 96.35,	Neg-Neu (*p* = 0.001),	*F*(2, 4902) = 11.93,
	*p* = 0.001, η2 = 0.327	*p* = 0.001, η2 = 0.038	Neg-Pos (*p* = 0.001),	*p* = 0.001, η2 = 0.005
			Neu-Pos(*p* = 0.001)	
Subjective Age of Acquisition	*F*(1, 4902) = 7.21, *p* = 0.007, η2 = 0.001	*F*(2, 4902) = 81.52, *p* = 0.001, η2 = 0.032	Neg-Neu (*p* = 0.001), Neg-Pos (*p* = 0.001), Neu-Pos(*p* = 0.001)	*F*(2, 4902) = 44.44, *p* = 0.001, η2 = 0.018

### Relations between measures

For all affective norms studies it is especially important to search for patterns in relations between assessed measures. Those relations, if repeatable across cultures and languages, can tell us more about the theoretical status of the affective meaning of stimuli. To check for a correlation pattern in the case of the ANPW_R dataset, *r*-Pearson correlation was applied in the case of affective, psycholinguistic and linguistic variables. The correlation pattern is presented in Table 6. To check the nature of inspected relations, additional regression analyses were conducted. In the Table 6, cases of higher value of variance explained by quadratic function are represented by lighter-shaded cells.

**Table 6:** **Correlations between the variables (***r***-Pearson's)**.

	**Arousal**	**Dominance**	**Origin**	**Significance**	**Concreteness**	**Imageability**	**Subjective age of acquisition**	**LN of frequency (subtlex_pl)**	**LN of frequency (Kazojć, 2011)**	**Number of letters**
Valence	–0.464^**^	0.693^**^	0.080^**^	0.163^**^	–0.078^**^	0.126^**^	–0.224^**^	0.126^**^	0.136^**^	–0.069^**^
Arousal		–0.135^**^	–0.460^**^	0.378^**^	0.378^**^	–0.176^**^	0.175^**^	0.055^**^	0.000	0.204^**^
Dominance			0.256^**^	0.224^**^	0.021	0.017	–0.081^**^	0.118^**^	0.119^**^	–0.048^**^
Origin				–0.272^**^	–0.299^**^	0.018	0.024	–0.021	–0.029^*^	–0.108^**^
Significance					0.685^**^	–0.448^**^	–0.043^**^	0.281^**^	0.252^**^	0.263^**^
Concreteness						–0.800^**^	0.287^**^	0.002	0.001	0.390^**^
Imageability							–0.515^**^	0.127^**^	0.133^**^	–0.324^**^
Subjective age of acquisition								–0.449^**^	–0.438^**^	0.310^**^
LN of frequency (subtlex_pl)									0.908^**^	–0.072^**^
LN of frequency, (Kazojć, 2011)										–0.097^**^

**Figure 4:**
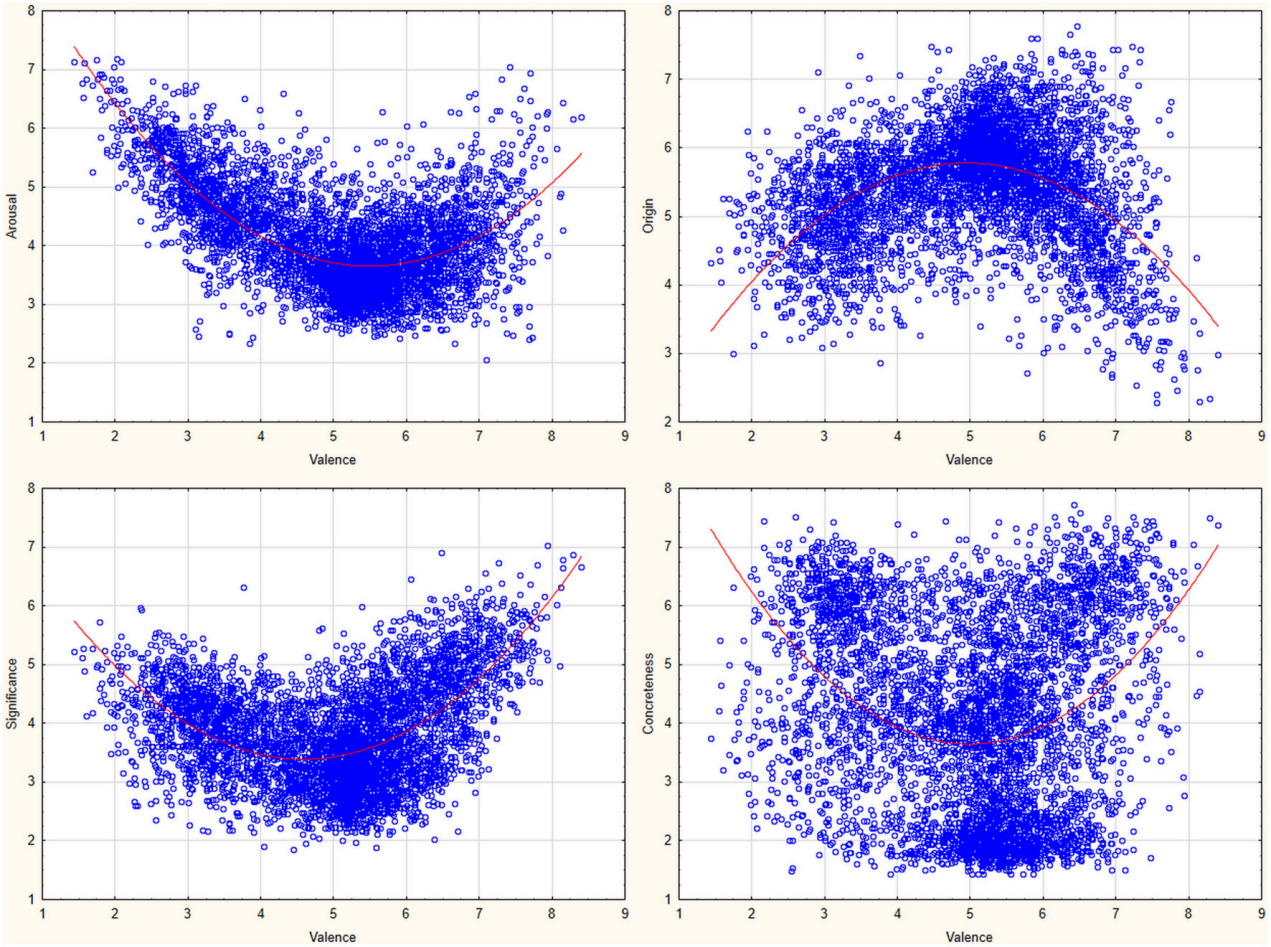
**Bimodal affective spaces distribution for dimensions correlated to valence in quadratic fashion**.

*Gray cells indicate quadratic relationship between measures rather than linear (higher R^2^ explained by quadratic function and significant R^2^change). For further information see Figure 4 and Figure 5 in Appendix 2 in Supplementary Material. LN, natural logarithm.*

* *p < 0.05;*

** *p < 0.001*.

Here only significant (*p* < 0.001) and large (*r* > 0.35, sharing more than 10% of common variance) correlations are discussed. It appears that valence correlates negatively with arousal (*r* = −0.464), which suggests that negative stimuli are more arousing than positive ones. It is quite a common finding that the valence and arousal relationship is quadratic in nature and forms a “U” shaped curve. For further investigation of this correlation the regression analysis with Valence as the independent factor and Arousal as the dependent factor was carried out to test both the quadratic and the linear models of the valence and arousal relationship. This analysis showed that the Valence and Arousal relationship in the ANPW_R is better explained by the quadratic function *y* = 0.227*x*^*2*^ − 2.493*x* + 10.503: *R*^*2*^ = 0.48, *F*_(2, 4902)_ = 0 2253.4, *p* = 0.001, rather than the linear relationship: *R*^*2*^ = 0.22, *F*_(1, 4903)_ = 1346.22, *p* = 0.001, which accounted for less variance. Also *R*^*2*^ change due to inclusion of the quadratic function was highly significant: *F*_(1, 4902)_ = 2478.4, *p* = 0.001. Figure 4 presents the dimensional distributions of ratings as well as best fitting to the data function.

Taking into account affective variables, dominance is highly positively correlated with valence (*r* = 0.693), which means that positive words are perceived as evoking controllable experiences, while negative as uncontrollable ones. Arousal is negatively correlated with origin (*r* = −0.46), which means that automatic-originated stimuli are more arousing than reflective-originated ones. Arousal is positively correlated with significance (*r* = 0.378), which suggests that more arousing stimuli are also perceived as more crucial and subjectively significant. Taking into account relations between affective or arousal and psycholinguistic measures, concreteness correlates positively with arousal (*r* = 0.378) and subjective significance (*r* = 0.685), which means that abstract stimuli are more arousing and subjectively significant than concrete ones. Imageability is negatively correlated with subjective significance (*r* = −0.448), which means that easier-to-imagine-words stimuli are perceived as less significant. Taking into account psycholinguistic variables, imageability is negatively correlated with concreteness (*r* = −0.8), thus easier-to-imagine-words stimuli are perceived as more concrete. Subjective age of acquisition assessments were negatively correlated with imageability (*r* = −0.515) and both frequency estimations (natural logarithms: LN) on the basis of the Subtlex_pl dataset (*r* = −0.449) and Kazojć (2011) dataset (*r* = −0.438). Those relations mean that words that are acquit later in an individual development are harder to imagine as well as less frequent. Also, concreteness was positively associated with length of words (*r* = 0.39), which means that abstract stimuli were composed of the larger number of letters in the ANPW_R dataset.

Additionally, in order to check the nature of relations between measures (liner or curvilinear), the regression analyses were conducted. Appendix 2 presents detailed results of regression analyses for cases when measures relation was better explained by a quadratic function (higher *R*^2^ explained by a quadratic function than a linear one and significant *R*^2^ change between functions—c.f. lighter-shaded cells in Table 6). All quadratic relationships found for valence are presented in Figure 4, while remaining are presented on Figure 5 located in Appendix 2 in Supplementary Material.

## Discussion

### Distribution, stability, and reliability of assessments

As shown in Figure 2, the assessments cover the whole scale for valence, concreteness, and dominance, while there was a relative lack of highly arousing, significant and acquired-later-in-age words as well as low imaginable and heart-originated ones. The valence distribution is very similar to that obtained in the original ANEW dataset (Bradley and Lang, 1999), other adaptations (Redondo et al., 2007; Soares et al., 2012; Montefinese et al., 2014) and norms for a greater number of words (Lahl et al., 2009; Moors et al., 2013; Warriner et al., 2013; Riegel et al., 2015). Mean and standard deviation distributions shown in Figure 3 indicate that for valence, dominance and partly origin there is a group of neutral/ moderate words that are perceived in an unambiguous way (low *SD*-values), but moderate values for other dimensions resulted from an ambiguous perception of affective reaction (high *SD*-values). Such findings are common in affective norms studies. For example, in ANEW (Bradley and Lang, 1999), NAWL (Riegel et al., 2015) and the Italian ANEW adaptation (Montefinese et al., 2014), neutral in the valence dimension stimuli, were composed of both low *SD* and high *SD* stimuli.

Split-half assessment shows that the current dataset provides highly reliable values for all measured dimensions that are comparable with other existing datasets (Redondo et al., 2007; Soares et al., 2012; Moors et al., 2013; Montefinese et al., 2014; Imbir, 2015a; Riegel et al., 2015). Fifteen doubled-words analyses also showed that assessments were reliable and stable within the current study. It is interesting to note that correlations with other existing Polish language datasets are very satisfactory. This is the case with valence, arousal, dominance, origin and significance for 1586 words reassessed from the ANPW (Imbir, 2015a) as well as 1274 words shared with the NAWL (Riegel et al., 2015). This means clearly that the method of assessment used (c.f. Moors et al., 2013) is as good as traditional paper-and-pencil (e.g., Imbir, 2015a) estimations collected from a large number of participants assessing a low number of words.

### Sex differences

Sex differences in affective reaction perception to words have been found several times in affective norms creation studies (c.f. Soares et al., 2012; Monnier and Syssau, 2014; Montefinese et al., 2014; Riegel et al., 2015). It is often expected, based on a stereotypical picture, that women are more emotional than men (e.g., Montefinese et al., 2014). Also, arousal and dominance are expected to be different between women and men in that men should perceive their reactions as more polarized in arousal and dominance (Montefinese et al., 2014). In the ANPW_R ratings between female and male participants they were found to correlate rather highly and were even comparable with levels of split-half estimation of reliability (c.f. Table 3). Using ANOVA analyses (c.f. Ferré et al., 2012; Monnier and Syssau, 2014) with data from the ANPW_R, all variables were found to differ for female and male ratings. In fact, women perceived valence in a more polarized way than men (c.f. Table 4), which means that negative words were more negative whereas positive ones were more positive in comparison to men's ratings. Arousal, dominance, origin, significance, imageability, and subjective age of acquisition dimension assessments were higher, while concreteness was lower for women than men, but not more polarized as it is in the case of valence. Interaction in the case of subjective age of acquisition revealed (c.f. Table 4), that negative words are perceived by men as learned earlier in comparison to women. Reversed relation can be observed in the case of positive words. Results for valence, arousal, dominance, and imageability are coherent with previous findings (Montefinese et al., 2014; Riegel et al., 2015).

### Relations between affective variables

The pattern of correlations presented in the ANPW_R is consistent with previous findings concerning affective norms for words. For example, valence and arousal were found to follow a quadratic relationship (e.g., Redondo et al., 2007; Soares et al., 2012; Moors et al., 2013; Monnier and Syssau, 2014; Montefinese et al., 2014; Imbir, 2015a; Riegel et al., 2015), meaning that for neutral words we observe a low arousal level, while for both negative and positive stimuli the arousal level is higher. Although this is a general trend, one may find words that do not follow this trend, and despite a neutral valence, are high- rather than not negatively low-arousing stimuli (c.f. Figure 4). Also, the arousal and dominance relationship appeared to be better described by the quadratic function. This was found earlier in the Italian adaptation of the ANEW list (Montefinese et al., 2014) and in Affective Norms for 718 Polish Short Texts (Imbir, submitted). This could be explained quite easily by the high positive correlation between valence and dominance, suggesting that both dimensions share much in common, thus correlating in a similar way with arousal.

In the ANPW_R dataset, six more quadratic relations were found to explain better the correlations between measured dimensions. For valence (c.f. Figure 4) those were the origin and subjective significance dimension cases. Taking into account origin, most valenced (negative and positive) words were perceived as more automatic-originated, while neutral was seen as more reflective-originated. This is probably because of the association of metaphors used to describe both ends of the origin scale. “Heart” is associated with passion and emotions, while “mind” is associated with reason and much less with passion. Similar results were found in the case of Polish Short Texts (Imbir, submitted). For significance, both ends of the valence scale were perceived as more crucial (subjectively significant) than neutral words. This is a similar pattern to that obtained in the valence and arousal case relationship in which valenced words were simultaneously more arousing ones. The previously mentioned arousal and dominance relationship can also be seen for subjective significance and dominance. The same moderate stimuli from the dominance scale are perceived as less subjectively significant in comparison to both controllable and uncontrollable stimuli. This could support the expectation that arousal and significance are two distinct mechanisms of activation interacting in a similar way with valence, but correlated with each other at a moderate level (*r* = 0.378).

The quadratic relation of origin and subjective significance was also found to be similar to the valence and arousal correlation. Moderate originated words were perceived as less significant than both automatic- and reflective-originated ones. In several cases of relations between valence and concreteness or imageability, arousal or dominance with concreteness, as well as origin and imageability, the distribution patterns shown on Figure 4 and Figure 5 in Appendix 2 in Supplementary Material are much less clear (c.f. Figure 4) and many exemptions from the general trend can be easily observed. The quadratic function still explains correlations better than the linear relation.

To sum up, the pattern of correlations results supports the claim that arousal and subjective significance are both activation aspects of affective reactions to stimuli. Also, valence and origin relate with both activation mechanisms in a similar way. The origin and valence relationship is challenging for the expectation of no relation between the two factors, but this is probably due to the metaphor used in the SAM scale construction. Dominance and valence are similar in relation to other dimensions, thus it is quite logical to omit dominance in affective norms creation (c.f. Riegel et al., 2015).

### Relations between affective and psycholinguistic variables

Relations between affective and psycholinguistic measures are also worth interpretation (Citron et al., 2014), since it is a relatively new part of affective norms studies. The ANPW_R, due to large number of assessed dimensions, gives us an opportunity for wide inspection of relations between two different types of measures. The results confirmed earlier findings for Spanish words (Hinojosa et al., 2016) that concreteness is negatively correlated with valence. The positive, linear correlation of concreteness and arousal was found in the ANPW_R. This result is coherent with Hinojosa et al. (2016), but not coherent with Italian norms (Montefinese et al., 2014) reporting quadratic relation between those measures. The relation between imageability and arousal was found to be quadratic in the ANPW_R, which is coherent with Montefinese et al. (2014), but different to the findings of Citron et al. (2014) for English words. Finally, the subjective age of acquisition relation to affective measures was found to be negative for valence, the same as in the Dutch normative study (Moors et al., 2013), negative for dominance which is opposite to Moors et al. (2013) findings, and negative for arousal, also opposite to the results of Citron et al. (2014). The pattern of relations described above does not allow us to draw conclusions, especially because the correlations between psycholinguistic and affective measures are typically low, thus although significant, they are rather weak (c.f. Janschewitz, 2008; Moors et al., 2013; Citron et al., 2014; Montefinese et al., 2014; Hinojosa et al., 2016).

### Current study limitations

It is worth highlighting that the current study has limitations. First of all the translation procedure employed, based on combined bilingual machine and human based steps may not be enough to compare the results in word-to-word comparison of assessments in cross cultural studies. Also, using the ANPW_R one had to watch out for the number of assessments done for each word, because some words scored lower than 50 of assessments, due to their unfamiliarity to the participants. Those words are included in the dataset in order to allow scientist include the familiarity scores in possible usages of the ANPW_R.

### Possible use of the ANPW_R

A research method of the Affective Norms of 4905 Polish Words Reload (ANPW_R) is important for the development of affective research in the Polish-speaking samples. It provides norms for eight different affective and psycholinguistic scales describing perception of reactions to the stimuli. Due to two new proposed dimensions introduced in the ANPW (origin and significance: Imbir, 2015a), the ANPW_R allows researchers to test hypotheses concerning the new developments in the field of affective sciences using the duality-of-mind approach. Also, the inclusion of three psycholinguistic variables (concreteness, imageability, and subjective age of acquisition) makes the ANPW_R dataset go beyond the standard approach in affective norm generation studies. Appendix 1 in Supplementary Material also presents measures of frequency based on two different Polish datasets (Kazojć, 2011; Mandera et al., 2014) as well as grammatical classes and length for each word. The dataset can be used without restriction by all scientists interested in: (1) searching for word processing mechanisms or (2) wanting to manipulate the affective state of an individual. As a supplement to this list a Polish Pseudo-word List was prepared recently (Imbir et al., 2015b), providing a list of 3023 pseudo-words generated from words used in the ANPW_R and complementary to them in length.

### Description of the database

The normative values of the Polish adaptation of affective norms are included in the Appendix to this article. In the first two columns, the full list of Polish words (4905) and their English translations is provided. Then, four lexical variables (two measures of frequency in the Polish language, parts of speech, and number of letters) are presented. Starting from column H, five affective dimensions (valence, arousal, dominance, origin, and significance) as well as three psycholinguistic dimensions (concreteness, imageability and subjective age of acquisition) are reported. For each variable, the number of participants assessing single words [*N*], the range, represented by the minimal [*Min*] and maximal [*Max*] rates, the mean [*M*], and standard deviation [*SD*] are presented in subset columns of a dataset spreadsheet. The ANPW_R is freely available to the scientific community for noncommercial use as a form of supplemental online material.

## Author contributions

The author confirms being the sole contributor of this work and approved it for publication.

## Funding

The project was funded by the National Science Center on the basis of decision 2013/09/B/HS6/00303.

### Conflict of interest statement

The author declares that the research was conducted in the absence of any commercial or financial relationships that could be construed as a potential conflict of interest.
