# Corrigendum: Affective Norms for 4900 Polish Words Reload (ANPW_R): Assessments for Valence, Arousal, Dominance, Origin, Significance, Concreteness, Imageability and, Age of Acquisition

**DOI:** 10.3389/fpsyg.2021.707540

**Published:** 2021-08-25

**Authors:** Kamil K. Imbir

**Affiliations:** Faculty of Psychology, University of Warsaw, Warsaw, Poland

**Keywords:** affective norms, duality of emotion, duality of activation, polish language, psycholinguistic indexes

In the original article, there was a mistake in Supplementary Material Data Sheet 1 as published. Incorrect values were matched with 193 words (out of 4,905), also two phrases were missing from the table, that were included in the original study, namely *na zewnatrz* (*outside*) and *z powrotem* (*back*). This issue affected all variables in the dataset, however, the data analysis was based on a correct dataset, as the errors only occurred during the preparation of the Data Sheet for the publication. Below are listed all of the errors in previous version of the appendix to ANPW_R database, the numbers are based on the original numbering in the published appendix.

135. bez emocji (unemotional) - 171. bezwzgledny (merciless)

569. dom strachów (haunted house) - 570. dominujacy (dominant)

1,145. kara śmierci (death penalty) - 1,149. karaluch (cockroach)

1,345. kosz na śmieci (trash can) - 1,347. koszmar (nightmare)

1,767. młot kowalski (sledgehammer) - 1,768. młotek (hammer)

1,916. nazwa (name) - 1,932. nie (not)

1,933. nie lubić (to dislike) - 1,977. nielubić (dislike)

2,399. pan młody (groom) - 2,403. panika (panic)

2,415. para wodna (steam) - 2,419. parasol (umbrella)

2,622. pod wrazeniem (impressed) - 2,667. podwórze (yard)

2,844. pół kwarty (pint) - 2,845. półka (shelf)

3,278. samo zadowolony (self-satisfied) - 3,292. sanki (sled)

4,778. zmierzać (to aim) - 4,813. zostawić (to leave)

The author apologizes for this error and states that this does not change the scientific conclusions of the article in any way. The original article has been updated.

## Publisher's Note

All claims expressed in this article are solely those of the authors and do not necessarily represent those of their affiliated organizations, or those of the publisher, the editors and the reviewers. Any product that may be evaluated in this article, or claim that may be made by its manufacturer, is not guaranteed or endorsed by the publisher.

