# Supplementary material for: Affective Norms for 4900 Polish Words Reload (ANPW_R): Assessments for Valence, Arousal, Dominance, Origin, Significance, Concreteness, Imageability and, Age of Acquisition
Source: Front Psychol. 2016 Jul 18;7:1081. doi: 10.3389/fpsyg.2016.01081 (PMC4947584; doi:10.3389/fpsyg.2016.01081)
Supplement: Supplementary file 2 [file DataSheet2.DOCX]

Appendix 2. Regression analyses results for curvilinear relations between variables in ANPW_R dataset.

**Quadratic Relations Involving Valence (Figure 4)**

In the case of valence and origin, the quadratic function *y* = – .2*x^2^* + 1.974 *x*+ .895 explained: *R^2^* = .23, *F*(2,4902) = 709.69, *p* = .001, while the linear relationship accounted for: *R^2^* = .006, *F*(1,4903) = 31.26, *p* = .001.*R^2^* change due to inclusion of the quadratic function was highly significant: *F*(1,4902) = 1379.3, *p* = .001.

For valence and subjective significance the quadratic function *y* = .237*x^2^*– 2.179*x* + 8.389explained: *R^2^* = .32, *F*(2,4902) = 1128.93, *p* = .001, while the linear relationship accounted for: *R^2^* = .029, *F*(1,4903) = 134.49, *p* = .001. *R^2^* change due to inclusion of the quadratic function was highly significant: *F*(1,4902) = 2066, *p* = .001.

In the case of valence and concreteness, the quadratic function *y* = .29*x^2^*– 2.898*x* + 10.876explained: *R^2^* = .123, *F*(2,4902) = 343.52, *p* = .001, while the linear relationship accounted for: *R^2^* = .006, *F*(1,4903) = 29.82, *p* = .001. *R^2^* change due to inclusion of the quadratic function was highly significant: *F*(1,4902) = 653.26, *p* = .001.

**Quadratic Relations Involving Arousal, Dominance and Origin (Figure 5)**

Another example is the case of the dominance and arousal relationship, where the quadratic function *y* = .194*x^2^*– 2.039*x* + 9.234explained: *R^2^* = .12, *F*(2,4902) = 317.57, *p* = .001, while the linear relationship accounted for: *R^2^* = .02, *F*(1,4903) = 91.57, *p* = .001.*R^2^* change due to inclusion of the quadratic function was highly significant: *F*(1,4902) = 533.62, *p* = .001.

Also, imageability and arousal is better explained by the quadratic function *y* = -.126*x^2^* + 1.428*x* + .274: *R^2^* = .078, *F*(2,4902) = 207.02, *p* = .001, rather than the linear *R^2^* = .031, *F*(1,4903) = 157.44, *p* = .001 function. *R^2^* change due to inclusion of the quadratic function was significant: *F*(1,4902) = 248.64, *p* = .001.

In the case of dominance and significance the quadratic function *y* = .235*x^2^*– 2.134*x* + 8.35 explained: *R^2^* = .191, *F*(2,4902) = 579.78, *p* = .001, while the linear relationship accounted for: *R^2^* = .05, *F*(1,4903) = 260.78, *p* = .001. *R^2^* change due to inclusion of the quadratic function was highly significant: *F*(1,4902) = 854.1, *p* = .001.

For dominance and concreteness measures, the quadratic function *y* = 396*x^2^*– 3.881*x* + 13.227 explained: *R^2^* = .108, *F*(2,4902) = 298.67, *p* = .001, while the linear relationship accounted for: *R^2^* = .001, *F*(1,4903) = 2.22, *p* = .136. *R^2^* change due to inclusion of the quadratic function was highly significant: *F*(1,4902) = 594.04, *p* = .001.

In the case of the origin and significance dimensions, the quadratic function *y* = .336*x^2^*– 3.802*x* + 13.227explained: *R^2^* = .221, *F*(2,4902) = 694.18, *p* = .001, while the linear relationship accounted for: *R^2^* = .074, *F*(1,4903) = 391.24, *p* = .001. *R^2^* change due to inclusion of the quadratic function was highly significant: *F*(1,4902) = 923.51, *p* = .001.

In the last case of quadratic relations found for origin and imageability, the quadratic function *y* = -.184*x^2^*+1.951*x* + 1.244explained: *R^2^* = .016, *F*(2,4902) = 40.05, *p* = .001, while the linear relationship accounted for: *R^2^* = .0001, *F*(1,4903) = 1.51, *p* = .22. *R^2^* change due to inclusion of the quadratic function was significant: *F*(1,4902) = 110.03, *p* = .001.

*
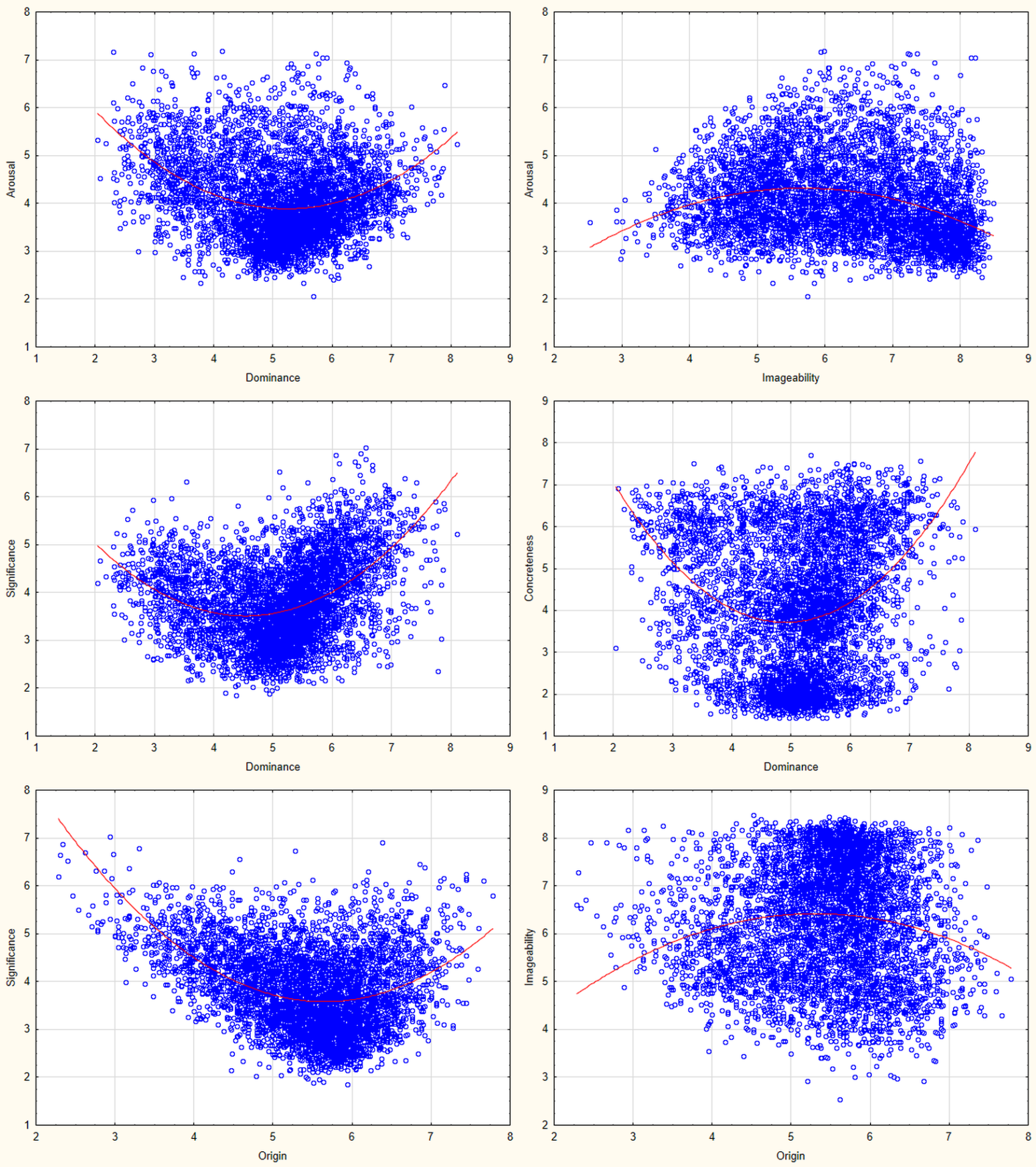
Figure 5.* Bimodal affective spaces distribution for six dimensions correlated to arousal, dominance and origin in quadratic fashion.
